# Surgical Experiences in the Present War

**Published:** 1915-01-23

**Authors:** 


### SURGICAL EXPERIENCES IN THE PRESENT WAR.

**(Contributions to this Section are Invited.)**

### Anti-Typhoid Inoculation in the Field.

BY AN OFFICER OF THE R.A.M.C. AT THE FRONT.

Since the "deadlock" in Flanders became so definitely established that neither side is able to advance anywhere more than a few hundred yards at a time, and very seldom even that, the Royal Army Medical Corps with the British Expeditionary Force has been concerned for the first time in the campaign much more with medical than with surgical work.

### Medical Work "in a Vast Bog."

Of this medical work a great deal consists in the care of those soldiers whose health or fighting efficiency becomes impaired through the rigours of an exceptionally wet winter in a country which is practically a vast bog. Another important aspect of the work is that of preventive medicine. Both French and Belgian Flanders are districts where the rudiments of hygiene and sanitation are apparently unknown, at all events, they are very little practised. When into such a country, at such a time of year, enormous numbers of troops are imported and billeted among villages and small towns, expert sanitation becomes absolutely necessary if disastrous epidemics of sickness are not to break out. This fact is well recognised by the heads of the Army—thanks largely, no doubt, to the lessons learnt at Bloemfontein in April and May 1900—and everything is being done, within the limitations imposed by the nature of the case, to diminish and prevent epidemic sickness. There have been, certainly, one or two small outbreaks in particular regiments of measles, mumps, and scarlet fever, contracted, in the latter case at least, through the failure of the local civilian authorities to notify the British military commander of the fact that a certain school which he allotted as a billet to some troops had been closed not long before owing to that very disease, and had not thereafter been properly disinfected. But in every such case prompt quarantine measures have soon suppressed the epidemics, none of which has been of any serious extent, so far as the present writer is aware.

There is, of course, one zymotic fever which has always been *par excellence* the scourge of field armies, more especially of armies not on the march —to wit, typhoid or enteric fever. In recent years the incidence of this disease, thanks to constant care and improvements in sanitation, including water supply, supervision of milk supplies, and other measures, such as bed isolation in hospitals, has been steadily reduced in Great Britain. With occasional exceptions, of which that at Lincoln in 1905 was perhaps the most considerable in quite recent years, typhoid fever has ceased to be epidemic in England, and its endemic distribution has been enormously curtailed. Hence among Englishmen of military age there are few who are protected against this disease by the artificial immunity of a previous attack.

These men enter the Army from an environment wherein—at least so far as typhoid fever is concerned—modern sanitation has cut down to a minimum their chances of acquaintanceship with the typhoid bacillus. The fate of war transports them to a region where every conceivable factor, barring high temperature and winged insects, is working to effect the introduction. Typhoid fever is endemic among the civil population, to whom the very elements of sanitation are unknown. The troops are billeted in a country of small farms and rather mean villages where their presence intensifies quite threefold the overcrowding which certainly exists even in peace time. Uncontaminated water there is none anywhere; such a thing as a water supply of decent bacteriological purity simply does not exist. The exposure inevitable in trench warfare, when every trench is half full of water or mud, is bound to reduce the natural resistance of the system to infection imported from without.

### Sanitation and Inoculation.

What, then, can be done to reduce the risk of an epidemic such as would endanger the fighting value of the British Expeditionary Force? Enforcement of sanitary regulations will, of course, help, and, in fact, has helped. But sanitation in these circumstances is at such an initial disadvantage that implicit trust cannot be placed in its effects. The other preventive measure that can be resorted to is preventive anti-typhoid inoculation, as practised in the British Army in India for a number of years. The results there achieved have been so beneficial that typhoid fever has been almost abolished as a source of mortality among the British garrison. The experience of several years has enabled the Army medical authorities to speak with confidence on the best methods of applying it, and of its enormous value both in preventing the fever and in modifying its virulence.

Accordingly, it has seemed good to those who control the Expeditionary Force to ordain that every officer and man shall be inoculated against typhoid fever; and this winter season of enforced comparative idleness is being taken advantage of to carry out the inoculation of all those who were not thus treated before they left England. It is, of course, impossible to assess at present the results of this attempt to control typhoid fever; indeed, it will probably be long after the end of the war before the statistics can be analysed and the success achieved can be estimated. All that the present writer can do is to offer a few comments, not very relevant perhaps to the scientific side of the matter, on anti-typhoid inoculation as it is being put in practice.

### The Process of Inoculation.

The system adopted is that of a small initial dose of anti-typhoid vaccine, followed ten days to a fortnight later by a larger one. The immediate effect of the first dose will be apparent within twenty-four hours, and often within six or eight. In a fair number of cases this is nothing beyond a slight stiffness or soreness of the arm (or other inoculated site) which soon passes off. In the majority of cases there is a slight malaise, with partial loss of appetite, but not sufficient to disqualify the subject from light work. In other cases the reaction is rather more severe, amounting to a moderate degree of pyrexia, with sufficient prostration to require twenty-four hours' complete rest. It may be laid down that within forty-eight hours, and usually in a much shorter time, the reaction from the initial dose has quite disappeared. It is stated that a brisk walk immediately following the injection will diminish the intensity of the subsequent reaction. Occasionally there is some degree of redness and swelling in the arm all round the site of inoculation; but it seems to be very exceptional for the axillary glands to enlarge. The present writer has not yet seen or heard of any such glandular swelling, nor any septic complication such as local abscess. The reaction to the second dose is usually *nil*, so far as the sensations of the recipient go; and in any case it is always less than that following the first dose.

In one case eleven out of thirteen men who were inoculated on a certain Thursday afternoon came back on the following Monday morning complaining of diarrhœa and vomiting, which began in every case on the Sunday evening or during the early hours of Monday. They had, as a group, for the most part had mild reactions, from which they had all recovered by Saturday. Probably the illnesses of Sunday night were of dietary origin, and not connected in any way with the inoculation.

### Conscientious Objectors.

Broadly speaking, there has been very little trouble with the conscientious objector, in spite of the lavish expenditure of certain anti-vivisection societies on full-page advertisements in the baser organs of the Radical press distributed free to the troops. For the vast majority the inoculation is a thing to be submitted to because it is an Order, and because Orders are things that have to be obeyed lest worse befall them. There have been sundry exceptions to this rule, some of them instructive, some humorous. The unexpressed objection of these recalcitrant soldiers is, beyond doubt, due to the extraordinary idea which has been spread about (by whom it is difficult to say) that anti-typhoid inoculation renders those who submit to it sexually impotent! Nor is it only among the rank and file that this extraordinary and totally unfounded conception has been spread about. Only a few hours ago a cavalry captain who came to be inoculated told the writer that a few days previously a staff officer had asked him whether it was true that this result occurred in 89 per cent. of cases!

The usual excuse for non-compliance given by the ordinary soldier is that he doesn't " 'old with it." One such objector told the writer that his three children had all been vaccinated, yet two of them had contracted typhoid fever. It was quite useless to point out to him that ordinary "vaccination" protects only against smallpox, and that his argument was, therefore, no argument at all. Another objector said he had been inoculated (against typhoid, of course) in India some years before, and within two years he "got malaria fever." Here, again, no explanations had any persuasive effect.

### Instances of Compulsion.

The Army Order commanding universal inoculation set forth the reason for which it was made compulsory: namely, that the object was rather the prevention of epidemics and the preservation of the fighting strength of the Army than the mere advantage of the individual himself. The latter effect does, naturally accrue also; but it is the safeguarding of the soldiers' comrades which makes compulsion necessary. It was also ordered, in one Army Corps at least, that any man refusing to be inoculated should be tried by court-martial for disobedience. This expedient has not often been necessary. In one case the officer in charge of a detachment knew that two of his men were determined objectors, and that they were doing their level best to induce the rest to refuse inoculation. When the time came for this unit to attend at a field ambulance for inoculation (of all those not already done before leaving home), he had the two ringleaders brought up first. Both refused to be inoculated, whereupon both were marched off, prisoners under close arrest. The remainder were so impressed by this drastic action that they one and all consented to be done. Next day the two prisoners were brought before their colonel, who gave them the option of court-martial for disobedience, or inoculation: they both chose the latter.

In another unit, where the officer commanding could not be induced by his attached medical officer to take any interest in inoculation or any other matter relating to the health of his troops, about forty out of two hundred declined the inoculation;* this is far and away the highest percentage of dissenters that the writer has so far encountered. In one unit, an infantry battalion, it is said that there were only two or three who refused; and they were forcibly held down and done under protest! This incident is only hearsay, and the writer cannot vouch for it. In the infantry only 10 per cent. of each battalion are allowed to be inoculated at any one time; and it is further laid down that no man is to be done within twenty-four hours of leaving the trenches, or within seventy-two hours of re-entering them. The reason of this very sensible regulation is self-evident.

* On a subsequent occasion about half of these men consented to be inoculated, and were then done. This was after the Army Order on the subject had been officially read out to them.

